# Evaluation of real-time PCR endogenous control genes for analysis of gene expression in bovine endometrium

**DOI:** 10.1186/1471-2199-10-100

**Published:** 2009-11-01

**Authors:** Caroline G Walker, Susanne Meier, Murray D Mitchell, John R Roche, Mathew Littlejohn

**Affiliations:** 1 DairyNZ Ltd., Hamilton, New Zealand; 2 Liggins Institute, The University of Auckland, Grafton, New Zealand

## Abstract

**Background:**

Quantitative real-time PCR gene expression results are generally normalised using endogenous control genes. These reference genes should be expressed at a constant level across all sample groups in a study, and should not be influenced by study treatments or conditions. There has been no systematic investigation of endogenous control genes for bovine endometrium to date. The suitability of both commonly used and novel endogenous control genes was evaluated in this study, with the latter being selected from stably expressed transcripts identified through microarray analysis of bovine endometrium. Fifteen candidate endogenous control genes were assessed across different tissue subtypes in pregnant and cycling Holstein-Friesian dairy cows from two divergent genetic backgrounds.

**Results:**

The expression profiles of five commonly used endogenous control genes (GAPDH, PPIA, RPS9, RPS15A, and UXT) and 10 experimentally derived candidate endogenous control genes (SUZ12, C2ORF29, ZNF131, ACTR1A, HDAC1, SLC30A6, CNOT7, DNAJC17, BBS2, and RANBP10) were analysed across 44 samples to determine the most stably expressed gene. Gene stability was assessed using the statistical algorithms GeNorm and Normfinder. All genes presented with low overall variability (0.87 to 1.48% CV of Cq). However, when used to normalise a differentially expressed gene (oxytocin receptor - OXTR) in the samples, the reported relative gene expression levels were significantly affected by the control gene chosen. Based on the results of this analysis, SUZ12 is proposed as the most appropriate control gene for use in bovine endometrium during early pregnancy or the oestrus cycle.

**Conclusion:**

This study establishes the suitability of novel endogenous control genes for comparing expression levels in endometrial tissues of pregnant and cycling bovines, and demonstrates the utility of microarray analysis as a method for identifying endogenous control gene candidates.

## Background

Quantitative real-time reverse transcription PCR (RT-PCR) is an extremely sensitive technique that allows the precise measurement of gene expression across more than seven orders of magnitude[1,2]. RT-PCR is often considered the gold standard for quantifying gene expression, and is commonly used to validate techniques with greater throughput but less overall sensitivity, such as microarray analysis [3-5]. RT-PCR relies on the use of fluorescent dyes to quantify transcript amplification, with the amplification cycle number at which these dyes/transcripts are detected (above background) giving an indication as to the relative abundance of the target molecules. The sensitivity of RT-PCR makes it a powerful tool for gene expression measurement, especially when sample quantities are limited or a transcript is expressed at a low level. However, this sensitivity also means that a great deal of care must be taken with regards to experimental design and implementation of the procedure.

When designing an experiment to evaluate gene expression in a group of samples, a number of critical factors must be kept constant. These include RNA extraction, DNase treatments, and cDNA synthesis. Normalisation of RT-PCR results is required to control inter-sample differences that may arise as a result of these sample processing steps, and ensure the gene expression of target transcripts are robustly quantifiable [6,7].

The most common method for normalising RT-PCR data involves the use of one or more endogenous control genes. An ideal endogenous control gene is one that is stably expressed within the samples to be compared, regardless of tissue differences, experimental conditions, or treatments. Choosing an endogenous control gene to normalise gene expression data is one of the most crucial steps in the experimental design. Genes used as endogenous controls in RT-PCR experiments are often chosen with little prior knowledge of their expression over the experimental conditions examined, and are often selected arbitrarily from a pool of commonly used endogenous control genes such as GAPDH, and β-actin [8]. The most widely used endogenous control gene in studies of endometrial gene expression is GAPDH [9-12]. However, the suitability of GAPDH as an endogenous control gene has recently come into question, especially due to its potential regulation in a wide variety of physiological states [13], making it a questionable choice for RT-PCR normalisation [14].

Over the past three decades, genetic selection for milk production has resulted in a significant decline in dairy cattle fertility [15]. The fertilisation rate in dairy cattle is around 90% and does not differ between low-moderate and high-producing animals. However, the calving rate in lower producing animals is approximately 55%, whereas in high-producing animals this rate is approximately 35%. Pregnancy losses are thought to occur primarily during the pregnancy recognition/pre-implantation period [16], making studies of endometrial gene expression critical to further understanding of pregnancy establishment, recognition and maintenance within the bovine reproductive cycle.

The primary aim of this study was to identify suitable endogenous control genes for analysis of endometrial tissues from pregnant and cycling bovines. This study also aimed to investigate the potential use of microarray data analysis for identification of novel endogenous control genes, and the effect of endogenous control gene selection on the calculated expression of a target gene.

A total of 15 candidate endogenous control genes were analysed in 44 samples representing two different tissues (intercaruncular and caruncular) from 22 animals. These animals were either pregnant or cycling at day 17 of the reproductive cycle, and represented Holstein-Friesian cows from two divergent genetic backgrounds (North American (NA), and New Zealand (NZ)).

Two strategies were employed to identify the candidates. Five genes were selected on the basis that they had been previously used as housekeeping genes [17-19], and an additional 10 novel genes were derived from a microarray experiment based around the same 44 samples used in the current analysis. Genespring GX software was used to generate a list through filtering on expression stability across the 44 samples. This list was subjected to GeNorm [20] and Normfinder [21] analysis to identify the 10 most suitable genes. The suitability of all 15 genes was then tested through statistical analyses, including a comparison of expression stability as determined by GeNorm and Normfinder algorithms. The effect of using these endogenous control genes was then evaluated using relative quantification of a gene known to be differentially expressed in the study.

## Results and Discussion

Microarray analysis of the 44 endometrial samples revealed 27 transcripts with a high degree of expression stability (filtering on expression level - upper limit 1.2, lower limit 0.833). GeNorm and Normfinder were utilised to identify the 10 most stably expressed transcripts for further analysis. For RT-PCR design, full length transcripts were identified by querying microarray probe sequences against the bovine genome (Btau3,1) using NCBI BLAST .

**Figure 1:**
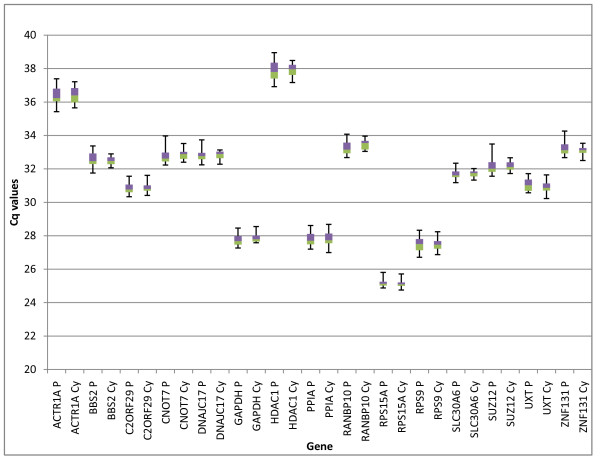
**Expression levels of candidate endogenous control genes in pregnant (P) and cycling (Cy) endometrial tissue samples**. Values are given as quantification cycle (Cq) numbers. Boxes represent the lower and upper quartiles with medians; whiskers illustrate the maximum and minimums of the samples. There were no significant differences (P > 0.05) between the Cq means (T-test) or variances (F-test) of pregnant and cycling animals for any genes tested (except the variance of BBS2, P = 0.039).

Gene expression levels of the candidate endogenous control genes (expressed in Cq values) are displayed in Table 1 and Figure 1. Cq values for sample replicates had very low variability with a mean intra-assay coefficient of variation (CV) of 0.41%. All genes had low overall variability, with the Cq range between 1.06 and 2.04 cycles, standard deviations ranging from 0.25 to 0.53 cycles, and CV values ranging from 0.87 to 1.48% Table 1.

**Table 1:** Quantification cycle (Cq) values and statistics for 15 candidate endogenous control genes assayed across 44 bovine endometrial samples.

**Gene Symbol**	**Mean Cq**	**std dev**	**%CV**	**Min Cq**	**Median Cq**	**Max Cq**	**Range Cq**
ACTR1A	36.40	0.53	1.45	35.42	36.27	37.39	1.97
BBS2	32.53	0.35	1.07	31.75	32.47	33.37	1.62
C2ORF29	30.84	0.30	0.98	30.34	30.78	31.62	1.28
CNOT7	32.80	0.39	1.19	32.23	32.76	33.97	1.74
DNAJC17	32.83	0.32	0.96	32.24	32.85	33.74	1.49
GAPDH	27.81	0.34	1.21	27.27	27.76	28.55	1.28
HDAC1	37.88	0.49	1.30	36.92	37.93	38.96	2.04
PPIA	27.81	0.41	1.46	26.99	27.72	28.68	1.69
RANBP10	33.36	0.37	1.11	32.68	33.33	34.08	1.40
RPS15A	25.16	0.25	1.01	24.75	25.12	25.81	1.06
RPS9	27.47	0.41	1.48	26.71	27.50	28.33	1.62
SLC30A6	31.70	0.28	0.87	31.18	31.67	32.34	1.16
SUZ12	32.16	0.39	1.21	31.56	32.10	33.49	1.93
UXT	30.99	0.35	1.12	30.23	30.96	31.72	1.49
ZNF131	33.17	0.36	1.08	32.50	33.12	34.26	1.76

Significance calculations between gene expression data for pregnant and cycling animals were performed on Cq and relative concentration values, as estimated through absolute quantification using the Roche LC480 software. No significant differences (P > 0.05) in means or variances (Cq or concentration) between pregnant and cycling endometrial tissues were apparent for any candidate genes (except BBS2, which had a p-value of 0.04 (F-test) for variance between pregnant and cycling animals). The means and variances between the two different strains of Holstein-Friesians also lacked significance (data not shown).

Cqs and relative concentration variances between the two different tissue types showed no significant differences, however means were significantly different for all genes, except RPS15A (*P *= 0.39 (T-test), *P *= 0.032 (ANOVA)).

The differences in means calculated between tissue subtypes likely reflect the distinct morphological and functional differences between caruncular and intercaruncular endometrium, which relate to their respective roles in reproduction. The 'caruncules' of the endometrium are specialised projections that are the site of embryo attachment. Caruncules become highly vascularised, and are the major site for small molecule and gaseous exchange. In comparison, intercaruncular tissue is highly glandular and responsible for early nourishment of the embryo through secretions of large molecules into the uterus [22-24]. The intercaruncular tissue is often thought to be more important in early pregnancy and, therefore, the majority of expression studies in pre-implantation bovine endometrium focus solely on intercaruncular tissue gene expression[25-27]. There is very little reported expression analysis of caruncular endometrium and no known studies comparing expression profiles of the two tissues.

### Expression stability testing of candidate genes

To further analyse the suitability of the candidate genes for use as endogenous controls for bovine endometrial tissues, expression stability was assessed using the GeNorm [20] and Normfinder [21] algorithms.

**Figure 2:**
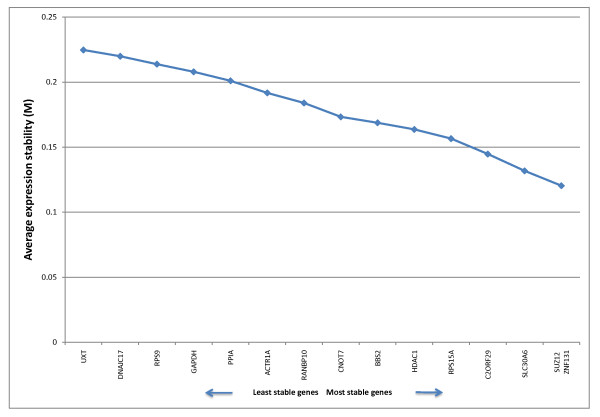
**GeNorm output. Average expression stability value (M) for candidate endogenous control genes in bovine endometrial tissue samples**. The M-value threshold for stability of a gene according to GeNorm is 1.5. The most stable genes as determined through analysis of the pairwise variation of each gene with all other genes were SUZ12 and ZNF131.

GeNorm rankings for the 15 genes tested are presented in Table 2 and Figure 2A. GeNorm identified SUZ12 and ZNF131 as the most stably expressed genes of the 15 candidates. Four of the five most stable genes were those derived from microarray data; the other was RPS15A, which was selected from the literature. By contrast, four of the five least stable genes were chosen from the literature, including GAPDH. The most stably expressed gene identified by Normfinder was SUZ12 (Table 2), which was also one of the two most stable genes identified in the GeNorm analysis. The best combination of genes identified by Normfinder was SUZ12 and C2ORF29. The five most stably expressed genes according to Normfinder consisted entirely of microarray-derived genes, while the least stable five contained three out of five genes selected from the literature, again including GAPDH. The comparative ranking of all genes for both GeNorm and Normfinder algorithm analyses is displayed in Table 2.

**Table 2:** Stability ranking of candidate endogenous control genes from Normfinder and GeNorm analyses.

	**Normfinder**		**GeNorm**	
**Rank**	**Gene Symbol**	**Stability**	**Gene Symbol**	**Stability**
1	SUZ12	0.043	SUZ12	0.12
2	C2ORF29	0.053	ZNF131	0.12
3	HDAC1	0.061	SLC30A6	0.132
4	SLC30A6	0.062	C2ORF29	0.145
5	CNOT7	0.066	RPS15A	0.157
6	ZNF131	0.071	HDAC1	0.164
7	BBS2	0.071	BBS2	0.169
8	RPS15A	0.077	CNOT7	0.173
9	RPS9	0.078	RANBP10	0.184
10	ACTR1A	0.08	ACTR1A	0.192
11	RANBP10	0.082	PPIA	0.201
12	GAPDH	0.083	GAPDH	0.208
13	UXT	0.084	RPS9	0.214
14	DNAJC17	0.085	DNAJC17	0.22
15	PPIA	0.086	UXT	0.225

Normfinder rankings of genes through analysis of inter- and intra group variability (groups defined include pregnancy status, tissue type and cow genetic strain). GeNorm rankings of genes based on pairwise analysis of expression stability.

GeNorm calculates an expression stability value (M) for each candidate gene based on pairwise comparisons of variability. Each gene is compared to every other gene to determine the two genes that contain the least variation. The stability value calculated for each gene is used to rank genes from least to most stable. The authors of the method give an M-value of 1.5 as a cut-off for suitability as an endogenous control gene. The principal behind the pairwise stability measure ranking is that two ideal candidate normalisation genes should have an equal expression ratio in all samples [28]. In the present study, all genes are well below the stipulated 1.5 M-value. The program then calculates a normalisation factor in each sample for the most stable genes (data not presented). GeNorm also calculates the optimal number of endogenous control genes to be used in the analysis of gene expression (Table 3). This value is determined by locating the point where addition of the next most stable gene does not significantly affect the normalisation factor, using a cut-off value of 0.15. In this study, the value for 2 genes was 0.042, suggesting that 2 genes should be sufficient to normalise the experimental data.

**Table 3:** GeNorm output used to determine the optimal number of endogenous control genes for normalisation in bovine endometrial tissue samples.

**V2/3**	**V3/4**	**V4/5**	**V5/6**	**V6/7**	**V7/8**	**V8/9**	**V9/10**	**V10/11**	**V11/12**	**V12/13**	**V13/14**	**V14/15**
0.04	0.03	0.03	0.02	0.02	0.02	0.02	0.02	0.02	0.02AAY	0.02	0.02	0.01

GeNorm calculates the minimum number of genes required for accurate normalisation. In this case, two genes were sufficient (V2/3 = 0.04). The number of genes necessary for accurate normalisation is defined as the point at which the addition of a gene has a non-significant effect on the calculation of the normalisation factor, the threshold for a non-significant difference being 0.15.

Normfinder is another freely available tool for the identification of stable endogenous control genes. The main point of difference between the two methods is that Normfinder takes into account both inter- and intra-group variability. The program not only identifies the most stable pair of endogenous control genes but also identifies the best overall endogenous control gene. The calculation of variability between groups is especially important in the present study considering the significant expression differences between the two tissue subtypes. The use of the two most stably expressed genes, in this case SUZ12 and C2ORF29 (Normfinder), should provide sufficient normalisation for tissue comparisons as Normfinder selects the best combination of genes whilst taking into account any grouping effects such as tissue type.

The differences in rankings of gene stability using the two algorithms could be due to the fact that they use very different methods to assess gene stability. GeNorm selects genes based on the pairwise variation between genes. The two most stably expressed genes are therefore those genes that share an expression profile. In contrast, Normfinder uses a model based algorithm that takes into account overall stability as well as any groups that may be present in the sample set. For example, if there are any grouping effects on gene expression a gene would be ranked lower than one that demonstrated variability not associated with any particular group.

### Effect of Endogenous control gene on target gene relative quantification

Temporal down-regulation of endometrial oxytocin receptor (OXTR) expression is a hallmark of early pregnancy, with embryonic interferon tau (IFNτ) thought to elicit this response [29,30]. Given the expectation of differential OXTR expression between pregnant and cycling animals, the effect of control gene stability on gene expression values of OXTR in the 44 endometrial samples was tested.

**Figure 3:**
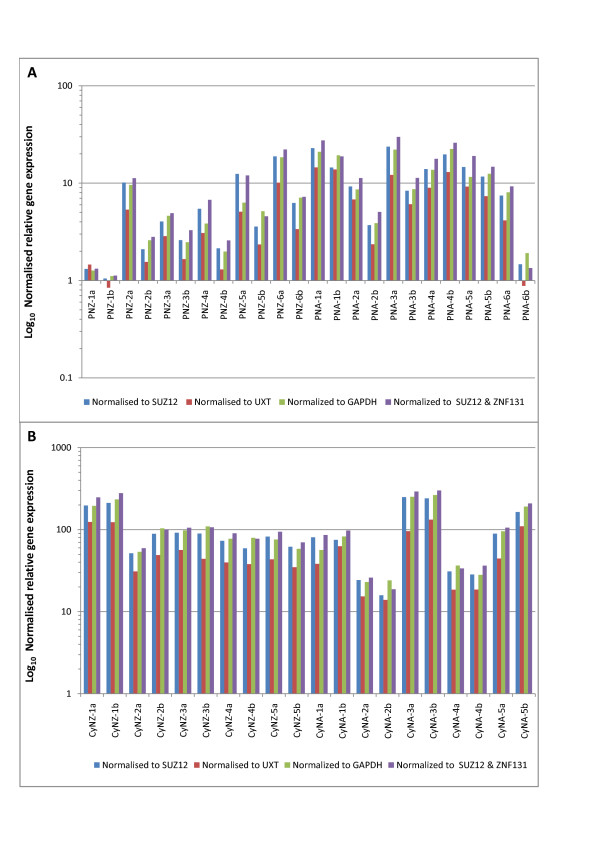
**Relative gene expression results for OXTR when normalised with endogenous control genes of different stability in pregnant (A) and cycling (B) animals**. These graphs show calculated relative expression values for OXTR when normalised to the most stable gene (SUZ12), two of the least stable genes (UXT and GAPDH), and to the two best genes according to GeNorm analysis (SUZ12 and ZNF131), with the latter using a normalisation factor calculated from the GeNorm analysis. (Pregnant = P, cycling = Cy, caruncular = a, intercaruncular = b, North American = NA, New Zealand = NZ)

Figure 3 presents relative OXTR expression levels when normalised with endogenous control genes of varying stability - the most stable gene identified by Normfinder (SUZ12), the two most stable genes identified by GeNorm (SUZ12 and ZNF131), and two of the least stably expressed genes identified by both Normfinder and GeNorm (GAPDH and UXT). Normfinder identified SUZ12 and C2ORF29 as the best combination of genes, but the relative expression was not significantly different from that calculated using only SUZ12 or a combination of SUZ12 and ZNF131 (data not presented). Correlation of normalised RT-PCR data to OXTR microarray expression data was not affected by choice of normalisation strategy. When compared to microarray reported expression, all calculated expression values had correlation coefficients of 0.79.

OXTR expression was significantly greater in cycling than in pregnant cows regardless of the endogenous control gene used (Figure 3, ANOVA, *P *< 0.01). Notably, OXTR expression in pregnant animals was greater on average and more variable in NA animals (which have lower fertility in general [31]) than for NZ animals (Figure 3A and 3B), and was also related to embryo size (data not presented). The use of different endogenous control genes had no effect on OXTR expression differences in these group comparisons.

However, there were differences in the normalised OXTR expression values that were inconsistent across the 44 samples, depending on which endogenous control genes were employed. For example, in sample Pregnant NZ-5a the calculated relative OXTR expression value was 12.46 when normalised to SUZ12, 6.32 when normalised to GAPDH, 5.10 when normalised to UXT, and 12.04 when normalised to both SUZ12 and ZNF131. Another sample (Pregnant NZ-2a), the values were 10.15, 9.60, 5.35, and 11.26 when normalised to SUZ12, GAPDH, UXT and both SUZ12 and ZNF131, respectively.

The average calculated fold change difference between pregnant and cycling animals was not affected by the choice of normalisation gene, possibly due to the large difference in expression level for this comparison (10-fold average difference between pregnant and cycling animals). However, the choice of reference gene could be important when normalising genes that exhibit more subtle variation between experimental groups, given the considerable variation in expression shown between individual samples.

## Conclusion

This study provides the first reported assessment of endogenous control genes for use in expression studies in bovine endometrium. Normalisation is a critical factor in reporting RT-PCR expression data, providing a necessary control for error associated with sample preparation. Normalisation using endogenous control genes provides a means of controlling this error, provided the gene(s) used are stably expressed across all samples under investigation. The study described here tested 15 candidate reference genes across 44 bovine endometrial samples representing a range of physiological states and tissue subtypes. This study evaluated the suitability of both commonly used and novel experimentally derived reference genes for use in normalisation of RT-PCR data. Candidates derived via microarray analysis were superior to existing, commonly used endogenous control genes, demonstrating the suitability of using microarray data for deriving novel endogenous control genes. This study also highlighted the importance of accurate normalisation with a stable endogenous control gene, by demonstrating relative expression of a differentially expressed gene when normalised using control genes of varying stability. SUZ12 was ranked first for stability across samples as determined by the statistical algorithms used in GeNorm and Normfinder, and is therefore proposed as the best gene for normalisation of RT-PCR data in the current study.

## Methods

All animal manipulations were carried out with the approval of the Ruakura Animal Ethics Committee (Hamilton, New Zealand). This work was conducted at No 5 Dairy, DairyNZ Ltd (Hamilton, New Zealand).

### Sample information

Endometrial tissue samples (intercaruncular and caruncular) were obtained immediately post-mortem from 22 Holstein-Friesian dairy cows. The exact details for sample information and collection are described in Meier et al 2009 [32]. Briefly, animals had estrous cycles synchronised, with half of the animals receiving a blastocyst stage embryo at day 7 of the estrous cycle. Endometrial samples were obtained post-mortem from each animal at day 17 of the reproductive cycle. The animals consisted of 12 pregnant and 10 cycling, further divided into North American (NA) and New Zealand (NZ) genetic ancestry (Table 4). This genetic strain model was chosen because NA cows have been reported to have poorer reproductive performance than NZ cows [31].

**Table 4:** Tissue categories: Two different tissue types each represented by four different groups of Holstein Friesians.

	**Pregnant**		**Cycling**	
	
**Tissue type**	**New Zealand**	**North American**	**New Zealand**	**North American**
**Intercaruncular**	6	6	5	5

**Caruncular**	6	6	5	5

### RNA Extraction

Endometrial tissue was homogenised in Qiagen buffer RLT (QIAGEN GmbH, QIAGEN, Hilden, Germany) using FastPrep Lysing Matrix D tubes in a FastPrep instrument (MP Biomedicals, Solon, OH).

Total RNA was extracted using a Qiagen RNeasy kit (QIAGEN). All samples were DNase treated using the Ambion DNA-free kit (Ambion, Austin, TX) according to the manufacturer's instructions. RNA quantity was determined by spectrophotometry in a Nanodrop ND-1000 (Nanodrop Technologies, Wilmington, DE). RNA integrity was checked using the Agilent 2100 Bioanalyzer with a RNA 6000 Nano LabChip kit (Agilent Technologies, Palo Alto, CA).

### cDNA Synthesis

One μg of an endometrial RNA sample was used for cDNA synthesis using the Invitrogen Superscript III Supermix kit (Invitrogen Corporation, Carlsbad, California). Total RNA was reverse transcribed according to the manufacturer's instructions using a final concentration of 27 μM of random pentadecamers primers. Briefly, RNA and random primers were mixed and denatured at 65 C for 5 minutes, followed by 1 minute on ice. Annealing buffer and Superscript/RNase was added to samples; these were then incubated for 10 minutes at 25 C (primer annealing), followed by 50 minutes at 50 C, and finally 5 minutes at 85 C to inactivate the enzyme. Reverse transcription (RT) negative controls were performed to test for the presence of genomic DNA contamination in RNA samples. Duplicate experimental samples were processed for cDNA synthesis as described above, but without the inclusion of the reverse transcriptase enzyme. Amplification was then tested for all genes using RT-PCR followed by assessment on a 3% agarose gel. No amplification was found in any of these samples.

### Candidate endogenous control genes

Fifteen potential endogenous control genes were selected either from a literature search or were identified from a microarray study through statistical analysis using Genespring GX (Agilent Technologies) software in combination with the GeNorm [20]and Normfinder [21] algorithms.

Briefly, Genespring GX software was used to analyse an array data set (Agilent 44k 60-mer oligonucleotide bovine array) representing 44 bovine endometrial samples collected as described above. All array data had undergone standard quality control and statistical analysis, including filtering on flags (present or marginal in all samples) and filtering on raw expression level of 200 to obtain reliable data. A list of 27 genes were derived from this dataset by filtering on normalised expression level (upper limit 1.2 and lower limit 0.833) to obtain genes that had stable expression across all 44 samples. This dataset was further analysed using the Microsoft Excel applets GeNorm and Normfinder. These programs were used to determine the 10 most stably expressed genes in the array data set.

### Quantitative Real-Time PCR

Real-time PCR using the Roche Lightcycler 480 (Roche) was performed on 15 candidate endogenous control genes for each of the 44 bovine endometrial samples using the Roche real-time PCR master mix (Lightcycler 480 Probes Master) in combination with Roche Universal Probe Library (UPL) assays. Assays were designed to publicly available bovine gene sequences (NCBI) using Roche UPL design software (ProbeFinder, v.2.45). All assays were designed to span an intron-exon boundary to prevent amplification of DNA. The primer and probe sequences are presented in Table 5.

**Table 5:** Characteristics of gene specific real-time PCR assays.

**Experimentally derived genes**						
**Gene symbol**		**Accession**			**Amplicon size (bp)**	**PCR efficiency**

DNAJC17	DnaJ (Hsp40) homolog, subfamily C, member 17	NM_001046276	Left primer	TCTGAAGATTTCCTGGTTGGA	90	1.92
			Right Primer	CTCTCGGACACCACTGAGC		
			Probe	#57: GGCCCCAG		

HDAC1	histone deacetylase 1	NM_001037444	Left primer	GGATGAGAAAGAGAAAGATCCAGA	76	1.53
			Right Primer	TTCTTGCTTCTCTTCCTTGGTT		
			Probe	#35:AGAAGAGGA		

RANBP10	RAN binding protein 10	NM_001098125	Left primer	CTCAACAGCGCCATTTTAGA	94	1.81
			Right Primer	CCATGAGCCGTAGACATTCA		
			Probe	#37:TGCCCTGG		

CNOT7	CCR4-NOT transcription complex, subunit 7	NM_001034312	Left primer	GATAGGACCGCAGCATCAG	111	1.82
			Right Primer	CCACAATATTTGGCATCATCA		
			Probe	#100:GCTCACAG		

ACTR1A	ARP1 actin-related protein 1 homolog A, centractin alpha (yeast)	XM_879421	Left primer	TATCTGCACCGCAGGAGAG	96	1.54
			Right Primer	CCTTCTTAGAGACCCACATCTTCT		
			Probe	#130:CTGGACAC		

BBS2	Bardet-Biedl syndrome 2	NM_001038160	Left primer	GAGCAGGTCATCTGCGTGT	132	1.90
			Right Primer	TCCCCTCCTAAGAAGAAGCTGT		
			Probe	#150:TGCTGTTC		

SUZ12	suppressor of zeste 12 homolog (Drosophila)	XM_582605	Left primer	GAACACCTATCACACACATTCTTGT	130	1.99
			Right Primer	TAGAGGCGGTTGTGTCCACT		
			Probe	#150:GAACAGCA		

ZNF131	zinc finger protein 131	NM_001101218	Left primer	AGAAAGAAGCTTTATGAATGTCAGG	94	1.90
			Right Primer	GTTTATCTCCAGTGTGTATCACCAG		
			Probe	#33:AGCTGGGA		

SLC30A6	solute carrier family 30 (zinc transporter), member	NM_001075766	Left primer	CAGTTGGACAAACTTATCAGAGAGG	66	1.90
			Right Primer	ATGCTCATTTCGGACTTCCA		
			Probe	#58:GGATGGAG		

C2ORF29	LOC506268 similar to Uncharacterized protein C2orf29	XM_582695	Left primer	CCTTCAAGAGCCCCCTGT	64	1.96
			Right Primer	GGGTCCTTTTCCAACTCTCC		
			Probe	#73:TCCTCAGC		

Literature derived genes						

**Gene symbol**		**Accession**				

RPS9	ribosomal protein S9	NM_001101152	Left primer	TGAGGATTTCTTGGAGAGACG	126	1.97
			Right Primer	ATGTTCACCACCTGCTTGC		
			Probe	#138:ATCCACCA		

UXT	ubiquitously-expressed transcript	NM_001037471	Left primer	AACTTCTTCGTTGACACAGTGG	130	2.01
			Right Primer	CTGTGAGGAGACTGCTCTTACG		
			Probe	#29:GGCAGAAG		

GAPDH	glyceraldehyde-3-phosphate dehydrogenase	NM_001034034	Left primer	GAAGCTCGTCATCAATGGAAA	67	1.94
			Right Primer	CCACTTGATGTTGGCAGGAT		
			Probe	#9:CATCACCA		

PPIA	peptidylprolyl isomerase A (cyclophilin A)	NM_178320	Left primer	GTCAACCCCACCGTGTTCT	99	1.94
			Right Primer	TTCTGCTGTCTTTGGAACTTTG		
			Probe	#152:GACGGCGA		

RPS15A	ribosomal protein S15a	NM_001037443	Left primer	TCAGCCCTAGATTTGATGTGC	104	1.85
			Right Primer	GCCAGCTGAGGTTGTCAGTA		
			Probe	#32:CTGCTCCC		

Gene of interest						

**Gene symbol**		**Accession**				

OXTR	Oxytocin receptor	NM_174134	Left primer	CGTGCAGATGTGGAGTGTCT	96	1.99
			Right Primer	TTGCAGCAGCTGTTGAGG		
			Probe	#162:TCCTGGC		

Roche Universal probe library assays were designed for 15 candidate endogenous control genes including 10 experimentally derived genes, five commonly used genes and one target gene - OXTR.

The PCR reaction volume was 10 μL consisting of 0.5 μM of each primer and 0.1 μM of probe. Standard cycling conditions were used [95 C for 10 minutes, (95 C for 10 seconds, 60 C for 30 seconds) × 50 cycles, 40 C for 40 seconds].

To quantify gene expression, cDNA was diluted 100-fold for all genes except ACTR1A, DNAJC17, HDAC1, RANBP10, and the target gene OXTR where cDNA was diluted 10-fold.

Each PCR run included a no-template control with water added instead of cDNA, as well as a RT negative control for each gene. Triplicate measurements were performed for all samples and standard curves. All samples for each gene were run on the same plate.

The Roche Lightcycler 480 software was used to perform quantification analysis of gene expression using the relative standard curve second derivative maximum analysis method, a non-linear regression line method. A six point relative standard curve of serial dilutions of cDNA was used with an estimated starting concentration of 1.0 and final concentration of 1.6E-03.

### Relative quantification of OXTR

The Roche Lightcycler 480 Software was used to perform advanced relative quantification analysis of OXTR gene expression. SUZ12, UXT and GAPDH were each used as a reference for the quantification of OXTR expression. Relative quantification was also performed using the normalisation factor of the two most stably expressed genes identified through GeNorm analysis (SUZ12 and ZNF131). Normalisation factors were calculated by taking the geometric mean of the two genes for each sample.

### Data analysis

Statistical analyses were performed using the Excel applets GeNorm (version 3.5)[20]and Normfinder [21] to estimate expression stability of the candidate endogenous control genes. GeNorm requires expression data to be input as concentrations determined via quantification, taking into consideration PCR efficiencies (Table 5). The program then estimates the most stable genes based upon pairwise comparisons of sample variability. The two most stable genes are identified and a normalisation factor calculated. Normfinder analyses the stability of the candidate genes taking into consideration inter-group variability. The program then ranks genes based on a stability value, with the lowest value indicating the most stably expressed gene. Significance tests were also performed on the data. Microsoft Excel was used to perform T-tests (with Bonferroni multiple testing correction applied) to test for significant differences between the means, and F-tests were used to assess differences in variance between experimental groups for each gene. ANOVA was also used to test for any significant difference in the means (GenSTAT). Pearson correlation calculation was used to assess the correlation of the microarray reported expression for the target gene (OXTR) and the RT-PCR reported expression.

## Authors' contributions

CGW was involved in experimental design, performed the experimental work and statistical analysis, and drafted the manuscript. SM designed the animal trial, carried out sample collection, project manager and funding of the project. JRR was involved in critical analysis of the manuscript and general supervision of the project. MDM was involved in critical analysis of the manuscript and general supervision of the project. MDL was involved in experimental design and critical analysis of the manuscript, and general supervision of the project.

All Authors read and approved the final manuscript.
